# From pixels to prognosis: Imaging biomarkers for discrimination and outcome prediction of pulmonary embolism: Original Research Article

**DOI:** 10.1007/s10140-024-02216-2

**Published:** 2024-03-25

**Authors:** Jennifer Gotta, Leon D. Gruenewald, Simon S. Martin, Christian Booz, Scherwin Mahmoudi, Katrin Eichler, Tatjana Gruber-Rouh, Teodora Biciusca, Philipp Reschke, Lisa-Joy Juergens, Melis Onay, Eva Herrmann, Jan-Erik Scholtz, Christof M. Sommer, Thomas J. Vogl, Vitali Koch

**Affiliations:** 1 Goethe University Hospital Frankfurt, Frankfurt am Main, Germany; 2 Department of Internal Medicine I, University Hospital Frankfurt, Goethe University, Frankfurt am Main, Germany; 3 Institut for Biostatistics and Mathematic Modelling, Goethe University Frankfurt, Frankfurt, 60590 Germany; 4 Clinic of Diagnostic and Interventional Radiology, Heidelberg University Hospital, Heidelberg, Germany; 5 University Hospital Frankfurt, Theodor-Stern-Kai 7, Frankfurt am Main, 60590 Germany

**Keywords:** Pulmonary embolism, Outcome, Dual-energy computed tomography, Survival, Prediction

## Abstract

**Purpose:**

Recent advancements in medical imaging have transformed diagnostic assessments, offering exciting possibilities for extracting biomarker-based information. This study aims to investigate the capabilities of a machine learning classifier that incorporates dual-energy computed tomography (DECT) radiomics. The primary focus is on discerning and predicting outcomes related to pulmonary embolism (PE).

**Methods:**

The study included 131 participants who underwent pulmonary artery DECT angiography between January 2015 and March 2022. Among them, 104 patients received the final diagnosis of PE and 27 patients served as a control group. A total of 107 radiomic features were extracted for every case based on DECT imaging. The dataset was divided into training and test sets for model development and validation. Stepwise feature reduction identified the most relevant features, which were used to train a gradient-boosted tree model. Receiver operating characteristics analysis and Cox regression tests assessed the association of texture features with overall survival.

**Results:**

The trained machine learning classifier achieved a classification accuracy of 0.94 for identifying patients with acute PE with an area under the receiver operating characteristic curve of 0.91. Radiomics features could be valuable for predicting outcomes in patients with PE, demonstrating strong prognostic capabilities in survival prediction (c-index, 0.991 [0.979–1.00], *p* = 0.0001) with a median follow-up of 130 days (IQR, 38–720). Notably, the inclusion of clinical or DECT parameters did not enhance predictive performance.

**Conclusion:**

In conclusion, our study underscores the promising potential of leveraging radiomics on DECT imaging for the identification of patients with acute PE and predicting their outcomes. This approach has the potential to improve clinical decision-making and patient management, offering efficiencies in time and resources by utilizing existing DECT imaging without the need for an additional scoring system.

**Supplementary Information:**

The online version contains supplementary material available at 10.1007/s10140-024-02216-2.

## Introduction

Pulmonary embolism (PE) represents a life-threatening condition caused by the sudden blockage of pulmonary arteries due to blood clots [1]. Given its diverse clinical presentations, accurate diagnosis and prognosis of PE remain sometimes challenging in clinical practices. With a high global prevalence, the complex nature of PE necessitates a nuanced approach to diagnosis and management. Traditional diagnostic methods, such as angiography and D-dimer assays, have demonstrated limitations in terms of sensitivity and specificity [2]. In this context, the integration of imaging biomarkers appears as a promising perspective to not only enhance diagnostic accuracy but also provide insights into individualized treatment approaches.

In recent years, imaging has emerged as a crucial approach for investigating PE with computed tomography pulmonary angiography (CTPA) serving as the established gold standard for diagnosis [3]. Advanced technologies offer the potential to extract specific biomarker-based information [4–6]. This development has heightened interest in the identification and validation of imaging biomarkers that not only contribute to the discrimination of PE but also aid in predicting treatment outcomes and prognostic parameters [7–9].

This study focuses on investigating the recent developments and advancements in dual-energy computed tomography (DECT) and radiomics for their utility in differentiating pulmonary embolism and predicting disease progression. Through the exploration of these modern technologies, our objective is to enhance the diagnosis and prognosis of PE, ultimately contributing to more effective patient management and care.

## Methods

The study was approved by the local ethical committee and was carried out accordingly, which exempted the need for obtaining written informed consent. All analyses were conducted in compliance with local data protection regulations.

### Study population

In this study, we compiled clinical data and CT datasets from a total of 131 patients who had undergone CTPA for suspicion of acute PE. The CT scans were performed using a third-generation dual-source dual-energy CT machine at the University Hospital Frankfurt (Frankfurt am Main, Germany). Data from this cohort have previously been reported [8]. The data collection period encompassed January 2015 to March 2022.

**Fig. 1:**
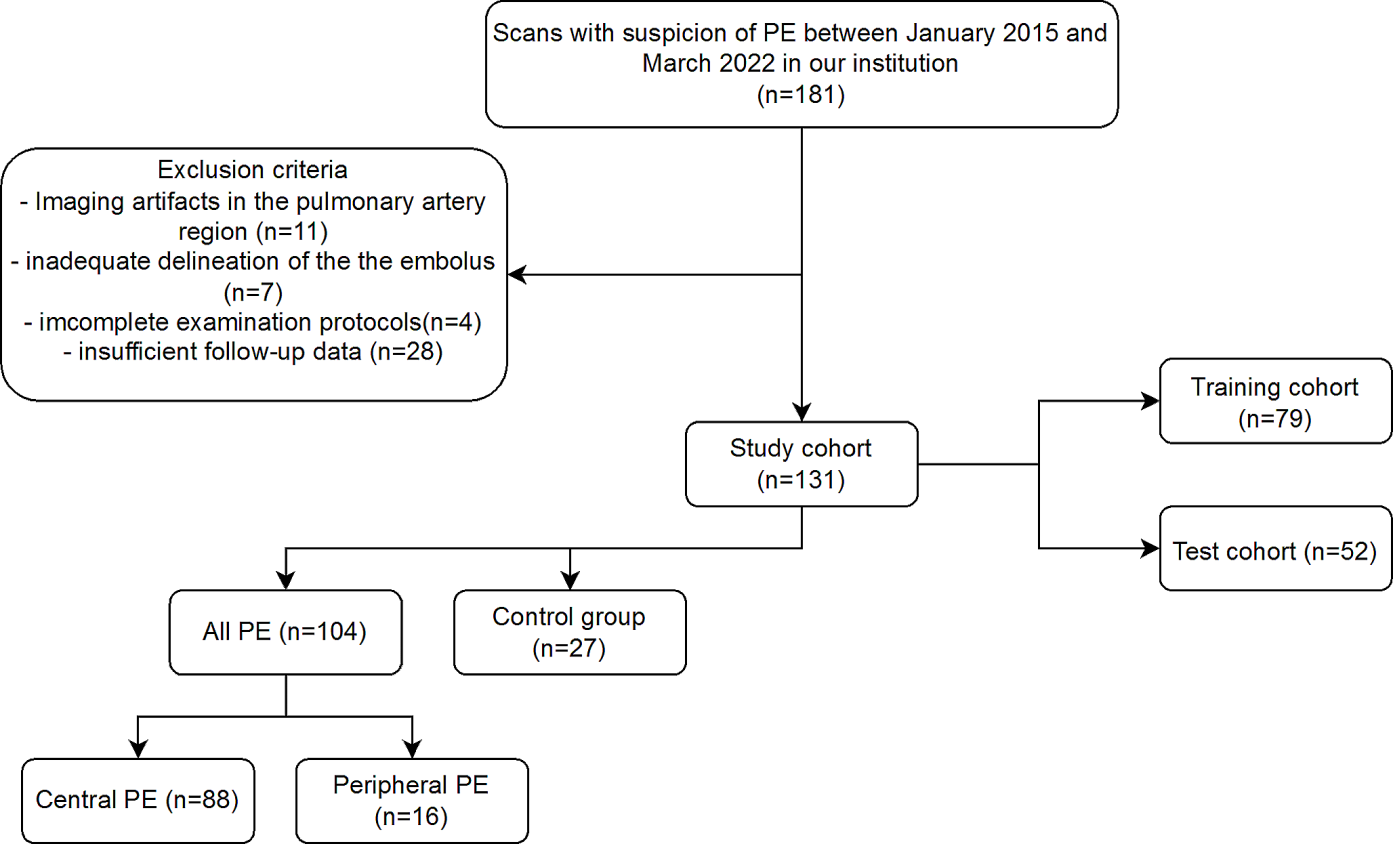
Flowchart of patient inclusion. *Abbreviations* PE, pulmonary embolism

All patients were divided into two distinct groups for analysis. One group consisted of patients with central PE and peripheral PE, while the other group who had no PE served as the control group. Inclusion criteria were patients over 18 years with a confirmed PE and the presence of a dedicated CTPA examination depicting the complete lung vasculature. Exclusion criteria in both cohorts encompassed imaging artifacts in the pulmonary artery region (n = 11), inadequate visual delineation of the embolus (n = 7), incomplete examination protocols (n = 4) and insufficient data on disease progression (n = 28). Eligible participants were identified from the picture archiving and communication system (Centricity™ RIS-i 7.0‘ [Version 7.0.3.5, 11/2021] und ‚Centricity™ Universal Viewer‘ [Version 7.0, 08/2021], General Electric Healthcare, Chicago, Illinois, USA) by conducting searches using specific terms such as ‘pulmonary embolism’, ‘pulmonal artery embolism’, ‘central pulmonary embolism’ and ‘peripheral pulmonary embolism’. The process of selecting participants for the study is illustrated in Fig. 1.

### Clinical and laboratory data

We obtained additional patient data and parameters by extracting information from medical reports and the hospital information system. Laboratory chemistry data were retrieved from recorded laboratory findings. The Wells and Pulmonary embolism severity index (PESI) scores were also calculated, further classifying patients into risk classes ranging from 1 to 5 based on the PESI score [2].

Additionally, a retrospective stratification of the patients into the four risk categories according the European Society of Cardiology (ESC) score was performed (low-risk PE, intermediate-low-risk PE, intermediate-high-risk PE, and high-risk PE) [2].

Overall survival was characterized as the duration from the DECT scan to the occurrence of death or the most recent documented follow-up.

### Dual-energy CT protocol

The CT scans were conducted using a third generation DECT scanner equipped with a Somatom Force unit manufactured by Siemens Healthineers (Forchheim, Bavaria, Germany).

The examination parameters were as follows: tube A, 90 kVp and 190 mAs; tube B, Sn150 kVp and 95mAs. In tube B, an additional tin filter (Selective Photon Shield II, Siemens Healthineers) was used to reduce radiation exposure. Acquisition parameters were 0.25 s (s) rotation time, 1–2 s acquisition time, 192 × 0.6-millimeter (mm) collimation, and 2.5 pitch value. Scanning was performed in the craniocaudal direction in bolus-triggered arterial and venous phases with 80–120 milliliters (ml) of nonionized contrast agent (Imeron 400, Bracco, Milan, Italy) at an injection rate of 5–6 ml/s, a threshold of 120 HU and a delay of 7s.

### Image segmentation and analysis

After anonymization, the CT datasets from all patients were extracted as Digital Imaging and Communications in Medicine (DICOM) datasets and uploaded into 3D Slicer (www.slicer.org, Version 5.0.2, Harvard University, Cambridge, USA).

**Fig. 2:**
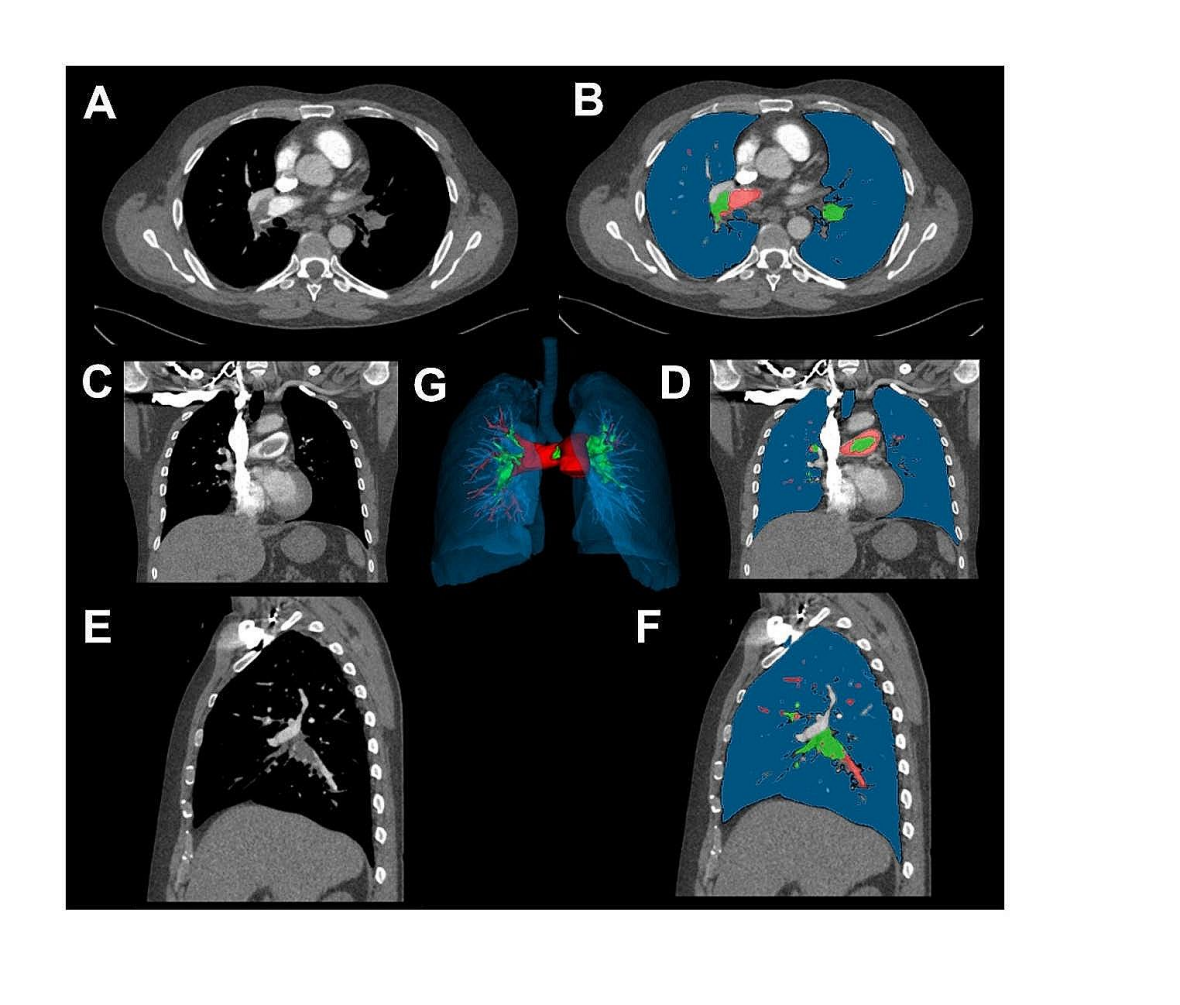
Exemplary segmentation of the pulmonary arteries and the embolus. **A**, Unsegmented axial slice; **B**, Segmented axial slice; **C**, Unsegmented coronal slice; **D**, Segmented coronal slice; **E**, Unsegmented sagittal slice; **F**, Segmented sagittal slice; **G**, Final segmentation of the embolus. *Abbreviations* PE, pulmonary embolism

The segmentation of the embolus and pulmonary trunk was performed in each patient using the interactive segmentation algorithm GrowCut [10–12]. An example of the segmentation is shown in Fig. 2. The segmentation in each case was evaluated by two experienced radiologist (V.K. and S.S.M.,  board-certified radiologists with four and seven years of experience in experimental imaging, respectively).

In case of any disagreement with the initial segmentation, the process was repeated, and necessary adjustments were made. All radiologists participating in the assessment were kept entirely unaware of the clinical data pertaining to the patients.

### Extraction of radiomics features

Radiomic features were extracted through the utilization of the PyRadiomics extension package incorporated into the 3D Slicer software (Version 5.1.0–2022-05–20). This extraction process generated a total of 107 features for each segmentation (Table S1) [13]. All extracted features were subsequently classified into seven distinct groups, as follows: Gray-Level Dependence Matrix (GLDM), Gray-Level Co-Occurrence Matrix (GLCM), Gray-Level Run Length Matrix (GLRLM), Gray-Level Size Zone Matrix (GLSZM), Neighboring Gray Tone Difference Matrix (NGTDM), Shape, and First Order [13].

To provide a comprehensive assessment of the study’s transparency and quality, we included a CheckList for EvaluAtion of Radiomics Research (CLEAR) [14] in Figure S1.

### Radiomics feature selection

In order to identify the most pertinent features for subsequent analysis, multi-stage feature selection process was implemented. Initially, all numerical features were normalized using Z-score standardization. In the next step, the Boruta dimension reduction and feature elimination algorithm, was applied [15]. Furthermore, a correlation analysis was conducted to identify clusters of highly correlated features, defined by a Pearson’s correlation coefficient (*r* ≥ 0.60). From each cluster, one feature with the highest Gini index was selected for further analysis.

### Construction of the radiomics model

A gradient-boosted tree model was trained on the selected radiomic features to discriminate between pulmonary embolism and healthy patients using a training dataset comprising 79 patients. The model was subsequently assessed using a distinct test dataset comprising 52 patients. This test dataset had not been incorporated into the algorithm’s training phase previously.

### Statistical analysis

Statistical analysis was conducted with the use of R statistical software (R Foundation for Statistical Computing, Vienna, Austria; Version 2023.06.0 + 421) and MedCalc (MedCalc Software Ltd., Ostend, Belgium; Version 20.123). The normality of the data distribution was assessed through visual methods such as histograms and the Wilk-Shapiro test. Normally distributed values were presented as mean ± standard deviation (SD), while non-normally distributed values were expressed as median and interquartile range (IQR). The t-test was employed for data with a continuous distribution, whereas the Man-Whitney test or Spearman rank correlation coefficient was applied to non-normally distributed data. A significance level of less than 0.05 was considered statistically significant.

The diagnostic accuracy of the optimal predictive parameters was evaluated using the area under the curve (AUC) derived from receiver operating characteristic (ROC) analyses. Subsequently, diagnostic sensitivity and specificity were computed.

Cox proportional hazards models were used to identify independent factors among clinical markers, imaging markers, and radiomics features. Multivariate Cox proportional hazards models were adjusted for significant univariate prognostic parameters and clinically relevant confounders, with hazard ratios and their corresponding 95% confidence intervals (CI) reported.

## Results

### Patient characteristics

A total of 181 patients were initially considered for study inclusion. After applying exclusion criteria, the ultimate study population encompassed a cohort of 131 patients aged 64 ± 15 years. Among these, 88 individuals (67%) received a final diagnosis of central PE, while 16 patients (12%) were diagnosed with peripheral PE. The comparative group comprised 27 patients who did not have PE at discharge. Table 1 provides an overview of the socio-demographic and clinical characteristics.

**Table 1:** Baseline characteristics of the study population

Variables - n (%) or mean (SD)	All PE (*n* = 104)	Central PE (*n* = 88)	Peripheral PE (*n* = 16)	No PE (*n* = 27)
Demographics				
Overall age (years)	64± 14	61 ± 15	67 ± 13	63 ± 17
Male sex (n)	60 (58%)	53 (60%)	7 (44%)	16 (59%)
Female sex (n)	44 (42%)	35 (40%)	9 (56%)	11 (41%)
**Laboratory parameters**				
D-dimers (ng/ml)	10,537 ± 2921	15,547 ± 1895	5527 ± 3947	-
Troponin T (pg/ml)	68± 83	90 ± 116	45 ± 49	-
NT-proBNP (pg/ml)	3550 ± 5364	5298 ± 8226	1802 ± 2501	-
**Vital signs**				
Heart rate (bpm)	92 ± 25	100.± 25	83 ± 23	71 ± 17
Respiratory rate (/min)	20 ± 7	21 ± 8	18 ± 6	14 ± 3
Systolic blood pressure (mmHg)	13 8± 29	134 ± 31	141 ± 26	140 ± 23
Diastolic blood pressure (mmHg)	78 ± 15	78 ± 17	78 ± 12	80 ± 13
Saturation of peripheral oxygen (SpO2, %)	92 ± 6	92 ± 5	91 ± 6	95 ± 2
Temperature (°C)	36.9 ± 0.8	36.8 ± 0.6	37 ± 1.0	36.6 ± 0.7
**Risk factors**				
Obesity (n)	37 (36%)	33 (36%)	4 (25%)	-
Atrial fibrillation (n)	11 (12%)	11 (12%)	0 (0%)	-
Cancer (n)	34 (33%)	28 (30%)	6 (38%)	0 (0%)
Diabetes Mellitus (n)	17 (16%)	15 (16%)	2 (13%)	7 (26%)
Arterial hypertension (n)	47 (45%)	40 (43%)	7 (44%)	18 (67%)
Current smoking (n)	15 (14%)	12 (13%)	3 (19%)	9 (33%)
**PE risk groups**				
Low risk group	9 (9%)	5 (6%)	4 (25%)	-
Intermediate low risk group	25 (24%)	18 (20%)	7 (44%)	-
Intermediate high risk group	62 (60%)	57 (65%)	5 (32%)	-
High risk group	8 (9%)	8 (9%)	0 (0%)	-
**Initial treatment**				
Lysis (n)	10 (11%)	10 (11%)	0 (0%)	-
Lysis in course (n)	6(7%)	6 (7%)	0 (0%)	-
Unfractionated heparin (n)	61(59%)	57 (65%)	4 (25%)	-
Low-molecular-weight heparin (n)	33 (32%)	23 (26%)	10 (62.5%)	-
Oral anticoagulants (n)	10 (10%)	7 (8%)	3 (18.8%)	-
**Hospital stay**				
Length of hospital stay from event (days)	10 ± 7	11 ± 7	8 ± 6	-
Complications (n)	24 (23%)	21 (24%)	3 (18.8%)	-
Recurrence PE (n)	4 (4%)	3 (3%)	1 (6.3%)	-
Death caused by PE(n)	2 (2%)	2 (2%)	0 (0%)	-

*Note* This is a sub analysis of a previous published study (8). Abbreviations: TAPSE, tricuspid annular plane systolic excursion; sysPAP, systolic pulmonary artery pressure; RV, right ventricle; LV, left ventricle; PE, Pulmonary embolism; PESI, Pulmonary Embolism Severity Index; NT-proBNP, N-terminal prohormone of brain natriuretic peptide

Wells Scores were found to be higher in the central PE patient group (*p* = 0.002), whereas no significant differences were observed for PESI (*p* = 0.7163). Significantly varying factors between central and peripheral PE patients included heart rate (*p* < 0.001), D-dimers (*p* < 0.0001), and CT-derived measurements, such as RV/LV-ratio (*p* < 0.0004) and truncus pulmonalis diameter (*p* < 0.0006).

### Selection of radiomics features

**Fig. 3:**
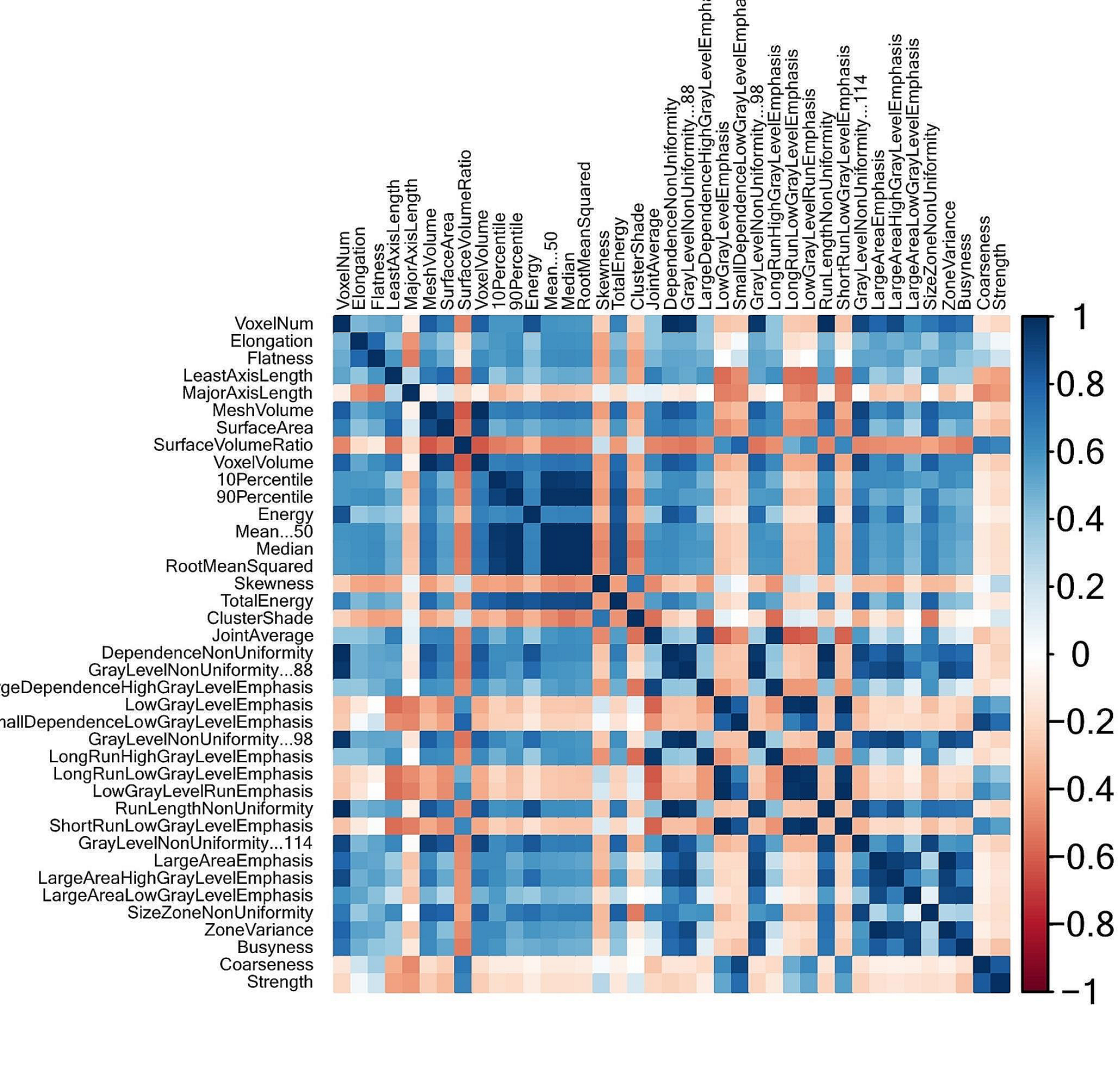
Correlation matrix. Correlation matrix of the most important features. *Abbreviations* PE, pulmonary embolism; GLCM, gray-level co-occurrence matrix; GLRLM, gray-level run length matrix; GLSZM, gray-level size zone matrix; NGTDM, neighboring gray tone difference matrix; GLDM, gray-level dependence matrix; MCC, maximal correlation coefficient

Following the implementation of the Boruta algorithm, 46 features were retained for subsequent analysis. In the subsequent step, a correlation matrix was generated. Figure 3 provides a visual representation of the correlation matrix, highlighting the crucial features for distinguishing between pulmonary embolism and normal pulmonary artery configurations. After feature reduction, 26 features remained for further analysis. The most significant features identified through the multi-step feature selection process are presented in Table 2.

**Table 2:** The most important radiomics features

**Features**	VoxelNum	ClusterShade
	Elongation	DifferenceVariance
	Flatness	LargeAreaHighGrayLevelEmphasis
	LeastAxisLength	LargeDependenceLowGrayLevelEmphasis
	MajorAxisLength	LowGrayLevelEmphasis
	GrayLevelNonUniformity	ZonePercentage
	SurfaceVolumeRatio	SmallDependenceLowGrayLevelEmphasis
	10Percentile	LongRunLowGrayLevelEmphasis
	RootMeanSquared	ZoneVariance
	Energy	LargeAreaLowGrayLevelEmphasis
	Skewness	SizeZoneNonUniformity
	Total Energy	SizeZoneNonUniformityNormalized
		Coarseness
		Strength

*Abbreviations* PE, pulmonary embolism; GLCM, gray-level cooccurrence matrix; GLRLM, gray-level run length matrix; GLSZM, gray-level size zone matrix; NGTDM, neighboring gray tone difference matrix; GLDM, gray-level dependence matrix; MCC, maximal correlation coefficient

### Performance of the radiomics model in detecting pulmonary embolism

In the next step, a gradient-boosted tree model was trained on these selected radiomic features to differentiate between pulmonary embolism and normal pulmonary artery configurations. To assess the model’s classification performance and its ability to generalize, we applied the trained model to an independent test dataset, encompassing 52 patients who were not part of the initial analysis. The classification accuracy in the validation dataset was 0.94 with an AUC of 0.91. Table 3 provides a comprehensive overview of the classification accuracy, sensitivity, specificity, and AUC.

**Table 3:** Performance of the radiomics model for the differentiation between patients with and without acute PE

	Validation cohort	95% CI of the validation cohort
AUC	0.91	0.76-1
Sensitivity	1	0.90-1
Specificity	0.89	0.63-0.95
Accuracy	0.94	0.88-1
PPV	0.98	0.78-1
NPV	1	0.92-1

*Abbreviations*: PE, pulmonary embolism; AUC, Area under the curve, PPV, positive predictive value; NPV, negative predictive value

### Radiomics features performance in predicting the prognosis of pulmonary embolism

The Cox regression analysis unveiled that the selected radiomic features provided independent prognostic insights into mortality with a c-index of 0.991 (95% CI, 0.979-1.0, *p* = 0.0001). Following the adjustment of the unadjusted radiomics model by incorporating imaging parameters and clinical parameters showed that the prognostic accuracy decreased. Table 4 provides a summary of the performance of various Cox regression models in predicting the outcomes of patients with PE incorporating radiomic features, imaging parameters and clinical parameters.

**Table 4:** Performance of different Cox-regression models to predict outcome using radiomics features and clinical parameters.

Model	Harrells c-index	95% CI of c-index	Chi-squared	*P* value
Unadjusted Model 1	0.991	0.979-1.000	63.056	**0.0001**
Adjusted Model 2	0.991	0.979-1.000	63.056	**0.0002**
Adjusted Model 3	0.989	0.973-1.000	48.131	**0.0104**
Adjusted Model 4	0.991	0.979-1.000	63.056	**0.0002**

Model 1: unadjusted radiomics model. Model 2: additionally adjusted by age. Model 3: additionally adjusted by troponin. Model 4: additionally adjusted by PESI-score

• Variables that did not reach univariate significance:

◦ D-dimers (p=0.5987), TAPSE (p=0.6815)

• Variables that reached univariate significance:

◦ Gender (p=0.0002), RV/LV-ratio (p=0.0002), tachypnoea (p=0.0387), age (p=0.0002), PESI (p=0.0002), Well-score (p=0.0002), hemodynamic instability (p=0.0002), signs of right heart strain (p=0.0002)

*Abbreviations* CI, confidence interval

## Discussion

In our study, we explored the potential of DECT-derived radiomics to enhance the diagnosis and prognostication of PE. A precise and reliable diagnosis of PE plays a pivotal role in the management of patients experiencing an acute event. Identifying individuals at high risk for adverse outcomes is crucial for prompt interventions and the application of suitable treatment strategies, effectively minimizing the risk of complications and potential harm [2, 9, 16].

There is a growing interest in integrating machine learning (ML) into various medical contexts, with the potential to alleviate physician workload and expedite the diagnostic process [9, 17]. ML possesses an advantage in managing extensive, intricate and diverse data types, enabling it to automatically handle large datasets. Moreover, it has the capability to provide data-driven insights and contribute to decision-making in a broad range of medical conditions, delivering advantages that go beyond saving time and resources. Over the past decade, a growing body of evidence has indicated the value of incorporating quantitative imaging biomarkers into established clinical decision-making models, enabling automated extraction of valuable imaging features from to diagnose a wide range of medical conditions or to gain tissue information [5, 18].

While past research has explored the capacity of radiomic features to differentiate between malignant and benign masses and predict outcomes across diverse cancer types [4, 19–21], there is limited information on techniques for forecasting outcomes in patients with PE. A previous study investigated the performance of distinct ML models based on clinical parameters to assess the diagnostic performance for the detection of PE [9]. The ML models based on clinical markers achieved superior diagnostic performance in diagnosing PE than classical clinical assessment models like the Wells score combined with D-dimer, the revised Geneva score with D-dimer, or the Years algorithm [9].

Another recent study has showcased the capability of models utilizing radiomic features extracted from DECT to classify patients with pulmonary embolism according to established clinical risk scores [8]. Our machine learning classifier attained a classification accuracy of 94% in identifying patients with acute PE, with an area under the receiver operating characteristic curve of 0.91.Furthermore, our model indicates that radiomic features could play a significant role in predicting outcomes for patients with PE. The inclusion of radiomic features demonstrated strong prognostic capabilities in predicting survival (c-index, 0.991 [0.979–1.00], *p* = 0.0001). Notably, the addition of clinical or DECT parameters did not enhance the predictive power. Even the PESI score, a well-established clinical scoring system for prognostic assessment, failed to augment the predictive strength in our study cohort [2, 22]. One reason for this could be that radiomics can more effectively characterize the various thrombus components that are not visible to the naked eye. Therefore, further investigations are necessary to evaluate the robustness and applicability of this approach, with a focus on its integration into clinical practice.

However, our study acknowledges several limitations that deserve consideration. First, this study was conducted at a single center. This decision was deliberate to minimize variabilities associated with scanners from different manufacturers or scanner generations. Secondly, due to the retrospective study design, inherent limitations may affect the reliability and generalizability of the findings. Lastly, our study yielded an acceptable radiomics quality score [14]. This score provides an overview of the transparency of the study and facilitates the repeatability and reproducibility of radiomics research. While comparable to previous radiomics studies, a higher radiomics quality score could have further enhanced the power of our findings.

Our study offers valuable insights into this innovative approach using radiomics for acute PE diagnosing and prognostication. Nevertheless, these limitations underscore the need for future investigations involving larger and more diverse patient cohorts. Further validation and seamless integration of this approach with established clinical tools have the potential to enhance patient outcomes and refine the delivery of patient care, ultimately advancing the field of risk assessment and patient management for individuals with acute PE. This, in turn, can lead to improved prognostication and better-tailored interventions, optimizing the overall management of patients with acute PE.

In conclusion, our study underscores the promising potential of employing radiomics on DECT imaging for the accurate detection and outcome prediction of patients with acute PE. This innovative approach has the capacity to significantly impact patient care by providing more accurate risk assessment and tailored management strategies.

### Electronic supplementary material

Below is the link to the electronic supplementary material.

Supplementary Material 1

## Data Availability

All data and information used are included in this manuscript.

## Abbreviations

PE: Pulmonary embolism
CT: Computed tomography
DECT: Dual energy computed tomography
AUC: Area under the curve
ROC: Receiver operating characteristic
GLDM: Gray-Level Dependence Matrix
GLCM: Gray-Level Co-Occurrence Matrix
GLRLM: Gray-Level Run Length Matrix
GLSZM: Gray-Level Size Zone Matrix
NGTDM: Neighboring Gray Tone Difference Matrix
ML: Machine learning
PESI: Pulmonary Embolism Severity Index

## Declarations

### Statistics and biometry

No complex statistical methods were necessary for this paper.
